# Impacto da Pandemia por COVID-19 nos Procedimentos Cirúrgicos de Dispositivos Cardíacos Eletrônicos Implantáveis em um Centro de Referência Terciário

**DOI:** 10.36660/abc.20201378

**Published:** 2021-10-06

**Authors:** Roberto Costa, Katia Regina da Silva, Sarah Caroline Martins Saucedo, Laisa Arruda Silva, Elizabeth Sartori Crevelari, Wagner Tadeu Jurevicius Nascimento, Thiago Gonçalves Silveira, Alfredo Fiorelli, Martino Martinelli, Fabio Biscegli Jatene

**Affiliations:** 1 Universidade de São Paulo Instituto do Coração Unidade de Estimulação Elétrica e Marcapasso São PauloSP Brasil Universidade de São Paulo Instituto do Coração - Unidade de Estimulação Elétrica e Marcapasso, São Paulo, SP – Brasil; 2 Universidade de São Paulo Faculdade de Medicina Hospital das Clinicas São PauloSP Brasil Universidade de São Paulo Faculdade de Medicina Hospital das Clinicas Instituto do Coração, São Paulo, SP – Brasil

**Keywords:** Desfibriladores Implantáveis, Marca-passo Artificial, Estimulação Cardíaca Artificial, Pandemia, COVID-19, Hospitalização, Contaminação, Qualidade de Assistência à Saúde

## Introdução

A pandemia da COVID-19 mudou o panorama da prática cirúrgica das diversas especialidades médicas no mundo inteiro.^1 - 4^ Mudanças nas rotinas dos serviços de estimulação cardíaca artificial foram apoiadas pelas sociedades médicas da especialidade, que estabeleceram recomendações para a definição da gravidade dos casos e da urgência dos procedimentos cirúrgicos.^5 , 6^

O objetivo do presente estudo foi avaliar o impacto das medidas implementadas para enfrentamento da pandemia nos procedimentos cirúrgicos realizados na área da estimulação cardíaca artificial, pela comparação dos dados obtidos durante o pico da pandemia aos do período imediatamente anterior. Para tanto, avaliamos o número de pacientes atendidos, o perfil clínico dos pacientes, o perfil dos procedimentos e a taxa de diagnósticos comprovados de COVID-19 nessa população.

## Métodos

Estudo prospectivo, aprovado pelo Comitê de Ética em Pesquisa do Hospital das Clínicas da Faculdade de Medicina da Universidade de São Paulo (Plataforma Brasil: 26587419.7.0000.0068).

Foram avaliados consecutivamente 557 pacientes submetidos a implante inicial ou reoperação de dispositivos cardíacos eletrônicos implantáveis (DCEI). Dados especificamente relacionados à COVID-19 foram adicionados para análise no estudo.

Em 21 de março de 2020, a direção do Instituto do Coração do Hospital das Clínicas da Faculdade de Medicina da Universidade de São Paulo (InCor-HCFMUSP) determinou medidas específicas para o enfrentamento da pandemia: isolamento dos pacientes com COVID-19, adiamento dos procedimentos eletivos e realização apenas de procedimentos urgentes para salvar a vida ou impedir o comprometimento hemodinâmico dos pacientes. Para tanto, os procedimentos foram classificados em urgentes ou eletivos ( Tabela 1 ), de acordo com as recomendações internacionais.^5 , 6^

**Tabela 1:** – Classificação dos procedimentos cirúrgicos para implante de dispositivos cardíacos eletrônicos durante a pandemia de COVID-19

Procedimentos Urgentes	Procedimentos Eletivos
**Implantes iniciais**	**Implantes iniciais**
MP por bloqueio atrioventricular avançado irreversível Doença do nó sinusal com sintomas graves ou pausas longas	MP por bloqueio atrioventricular não avançado estável Doença do nó sinusal pouco sintomática
CDI para prevenção secundária	CDI para prevenção primária
TRC para insuficiência cardíaca refratária grave	TRC em pacientes estáveis
**Trocas de geradores de pulsos**	**Trocas de geradores de pulsos**
MP ou CDI com mínima carga de bateria restante	MP ou CDI com mais de 6 semanas de bateria restante
**Troca de cabos-eletrodos**	**Mudança de modo de estimulação (upgrade)**
Disfunção de cabos-eletrodos em paciente dependente de MP ou que já recebeu terapia inapropriada do CDI	*Upgrade* para CDI somente em casos de prevenção secundária *Upgrade* para TRC somente em casos de insuficiência cardíaca refratária grave
**Extração de cabos-eletrodos**	**Extração de cabos-eletrodos**
Tratamento de infecção relacionada ao DCEI	Cabos-eletrodos não infectados à exceção de quando existe necessidade de obtenção de via de acesso para implantar eletrodo fundamental para o adequado funcionamento do DCEI

*CDI: cardioversor-desfibrilador implantável; DCEI: dispositivos cardíacos eletrônicos implantáveis; MP: marca-passo; TRC: terapia de ressincronização cardíaca.*

Os pacientes estudados foram hospitalizados a partir de atendimento na unidade de emergência do próprio hospital, por referenciamento externo ou pela análise da lista de espera cirúrgica. Os procedimentos cirúrgicos foram realizados segundo a rotina habitual. Todos os pacientes foram seguidos por 30 dias após a alta hospitalar. De acordo com o momento das operações, os pacientes foram agrupados em: Grupo 1 - período anterior à pandemia (1º janeiro a 20/março); Grupo 2 - período do pico da pandemia (21/março a 31/julho). Os dados foram coletados e gerenciados no software REDCap.^7^

Foram comparados os procedimentos realizados durante a pandemia com o período anterior por meio de análise univariada, adotando-se o nível de significância de 5%. Para a comparação de médias dos dois períodos empregou-se o teste t de *Student* e, quando a suposição de normalidade dos dados foi rejeitada, utilizou-se o teste não-paramétrico de Mann-Whitney. Para se testar a homogeneidade entre as proporções foi utilizado o teste qui-quadrado ou exato de Fisher.

## Resultados

Entre os 557 pacientes estudados, houve equilíbrio entre os sexos, predominância da raça branca (86,5%) e a média de idade foi 64,5 ± 20,4 anos. Durante a pandemia houve aumento significativo na taxa de pacientes com doença cardíaca estrutural (p=0,016), hipertensão arterial (p=0,047), fibrilação atrial (p=0,047), valvulopatias (p= 0,048) e neoplasias (p= 0,011) ( Tabela 2 ).

**Tabela 2:** – Características clínicas e cirúrgicas da população estudada

Características clínicas e cirúrgicas	Total (N= 557)	Grupo 1 Antes da pandemia (N= 253)	Grupo 2 Pico da pandemia (N= 304)	p
Sexo masculino, n (%)	281 (50,4)	135 (53,4)	146 (48,0)	0,210
Idade (anos)	64,5 ± 20,4	65,6 ± 19,7	63,6 ± 20,9	0,248
Classe Funcional (NYHA), n (%)				
I – II	453 (81,6)	212 (83,8)	241 (79,8)	0,378
III - IV	102 (18,4)	41 (16,2)	61 (20,2)	
Doença cardíaca estrutural, n (%)				
Apenas distúrbios da condução ou ritmo	204 (36,6)	109 (43,1)	95 (31,3)	0,016
Cardiopatia isquêmica	101 (18,2)	48 (19,0)	53 (17,4)	
Cardiopatia não-isquêmica	252 (45,2)	96 (37,9)	156 (51,3)	
Comorbidades, n (%)				
Hipertensão arterial	360 (64,9)	153 (60,5)	207 (68,5)	0,047
Diabetes mellitus	151(27,2)	66 (26,1)	85 (28,2)	0,587
2Fibrilação atrial	143 (25,8)	55 (21,7)	88 (29,1)	0,047
Valvulopatias	96 (17,3)	35 (13,8)	61 (20,2)	0,048
Insuficiência renal crônica	59 (10,6)	25 (9,9)	34 (11,3)	0,600
Neoplasia em tratamento atual ou recente	15 (2,7)	2 (0,8)	13 (4,3)	0,011
Procedimento realizado, n (%)				
Implantes iniciais	269 (48,3)	138 (54,6)	131 (43,1)	0,007
Reoperações	288 (51,7)	115 (45,4)	173 (56,9)	
Indicação - Implantes iniciais, n (%)				
Bradiarritmia sintomática	202 (75,1)	107 (77,5)	95 (72,5)	0,533
Profilaxia da morte súbita cardíaca	30 (11,2)	15 (10,9)	15 (11,5)	
Ressincronização cardíaca	37 (13,8)	16 (11,6)	21 (16,0)	
Indicação - Reoperações, n (%)				
Depleção natural do gerador de pulsos	192 (66,7)	72 (62,6)	120 (69,4)	0,204
Disfunção em cabos-eletrodos	44 (15,3)	16 (13,9)	28 (16,2)	
Mudança do modo de estimulação	25 (8,7)	11 (9,6)	14 (8,1)	
Infecção no DCEI	10 (3,5)	5 (4,3)	5 (2,9)	
Outras	17 (5,9)	11 (9,6)	6 (3,5)	
Tipo de DCEI, n (%)				
Marca-passo ventricular	76 (13,6)	21 (8,3)	55 (18,1)	0,007
Marca-passo atrioventricular	322 (57,8)	155 (61,3)	167 (54,9)	
Cardioversor-desfibrilador implantável ventricular	23 (4,1)	8 (3,2)	15 (4,9)	
Cardioversor-desfibrilador implantável atrioventricular	49 (8,8)	28 (11,1)	21 (6,9)	
Terapia de ressincronização cardíaca	82 (14,7)	37 (14,6)	45 (14,8)	
Remoção do DCEI	5 (0,9)	4 (1,6)	1 (0,3)	

*NYHA: New York Heart Association; DCEI: dispositivos cardíacos eletrônicos implantáveis.*

**Figura 1:**
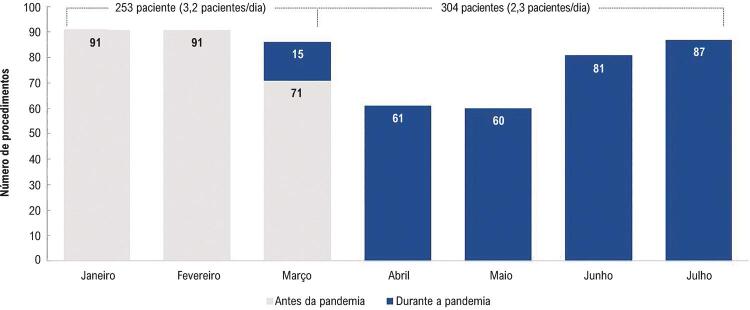
– *Número de pacientes submetidos a procedimentos cirúrgicos antes e durante o pico da pandemia da COVID-19.*

Durante o pico da pandemia, uma média de 2,3 pacientes foram operados por dia, em comparação a uma média de 3,2 pacientes / dia no período anterior, resultando assim, em redução de 27% no número de pacientes atendidos ( Figura 1 ). Houve modificação no perfil dos procedimentos, com predomínio de reoperações (p=0,070) e aumento da taxa de utilização de dispositivos unicamerais (p=0,007) ( Tabela 1 ).

O tempo de permanência hospitalar foi menor (p<0,001) durante o pico da pandemia, sem diferenças nas taxas de complicações pós-operatórias, readmissões ou mortalidade entre os grupos estudados ( Tabela 3 ).

**Tabela 3:** – Desfechos estudados no período pós-operatório de pacientes submetidos a implante de dispositivos cardíacos eletrônicos antes da pandemia da COVID-19 e durante o pico da pandemia

Desfechos do estudo	Total (N= 557)	Grupo 1 Antes da pandemia (N= 253)	Grupo 2 Pico da pandemia (N= 304)	p
Tempo entre a admissão hospitalar e o procedimento (dias)	5,4 ± 12,3	7,7 ± 15,3	3,5 ±8,7	<0,001
Tempo entre o procedimento e a alta (dias)	2,9 ± 7,3	3,5 ± 9,1	2,4 ± 5,4	<0,001
Tempo total de permanência hospitalar (dias)	8,3± 16,6	11,2 ± 20,7	5,9 ± 11,8	<0,001
Óbito hospitalar, n (%)	11 (2,0)	4 (1,6)	7 (2,3)	0,542
Óbito em 30 dias, n (%)	7 (1,3)	2 (0,8)	5 (1,6)	0,367
Readmissão hospitalar em 30 dias, n (%)	16 (2,9)	9 (3,6)	7 (2,3)	0,377
Reoperação relacionada ao DCEI, n (%)	21 (3,8)	10 (4,0)	11 (3,6)	0,837
Diagnóstico confirmado da COVID-19, n (%)	15 (2,7)	2 (0,8)	13 (4,3)	0,011

*DCEI: dispositivos cardíacos eletrônicos implantáveis.*

Um total de 15 (2,7%) pacientes tiveram o diagnóstico confirmado de COVID-19. Desses, 2 (0,4%) pacientes haviam sido operados antes da pandemia e permaneciam internados. Dos 13 pacientes operados durante a pandemia, 6 (1,1%) pacientes tiveram o diagnóstico de COVID-19 confirmado durante a hospitalização, e 7 (1,3%) durante os 30 dias que após a alta hospitalar.

Morte por qualquer causa foi observada em 18 (3,2%) pacientes, não tendo sido observada diferença significativa entre os grupos ( Tabela 3 ). Dos 15 pacientes que tiveram o diagnóstico de COVID-19, 7 morreram e, em todos eles, essa doença foi considerada a causa primária do óbito. Dentre as demais mortes ocorridas, a causa cardiovascular foi a mais frequente, responsável por 7 (1,3%) óbitos, seguida por infecção não relacionada ao DCEI em 2 (0,4%), complicação cirúrgica em 1 (0,2%) e neoplasia avançada em 1 (0,2%) caso.

## Discussão

Este estudo mostrou que as mudanças nas rotinas assistenciais permitiram o atendimento seguro e eficiente dos pacientes com indicação cirúrgica de estimulação cardíaca artificial durante a pandemia da COVID-19. Essas mudanças causaram, entretanto, redução no número de implantes iniciais e na complexidade dos dispositivos utilizados e aumento relativo nas trocas de gerador de pulsos por desgaste de bateria.

A despeito do aumento da gravidade dos pacientes atendidos durante a pandemia, o tempo de permanência hospitalar reduziu significativamente, tanto por redução do período pré-operatório quanto do período pós-operatório. Por outro lado, não houve diferenças significativas nos tipos de complicações relacionadas ao DCEI, na necessidade de reintervenções cirúrgicas ou na taxa de readmissões hospitalares nos 30 dias que se seguiram à alta, quando comparados os dois períodos estudados, ou quando comparado ao perfil histórico da instituição.^8^

Infelizmente, as medidas adotadas não foram suficientes para evitar a contaminação de pacientes pelo coronavírus, o que ocorreu em 2,7% dos pacientes do estudo. A falta de insumos para a aplicação de testes para detecção da COVID-19 pode ter causado subnotificação, como ocorreu em outras experiências publicadas,^9^ assim como a não identificação de indivíduos já infectados no momento da hospitalização. Pelo menos dois pacientes operados anteriormente à pandemia com internação prolongada apresentaram a doença. Dentre os operados durante a pandemia, seis pacientes manifestaram a COVID-19 durante a hospitalização e nos demais, o diagnóstico foi feito nos 30 dias que se seguiram à alta hospitalar, não sendo possível saber com certeza se a doença foi contraída durante a internação ou após a saída do hospital.

Embora sem significância estatística, a mortalidade total dos pacientes operados durante a pandemia foi maior em relação ao período anterior, e influenciada pela morte de sete dos 15 pacientes que contraíram a COVID-19 durante o estudo. A alta taxa de comorbidades dos pacientes que contraíram essa doença foi determinante para a evolução desfavorável deste subgrupo.

No momento atual, em que recomendações específicas para a retomada das atividades no período pós-pandemia começam a ser discutidas,^10^ a experiência ora reportada pode servir como embasamento para as atitudes que deverão ser tomadas numa possível recorrência do pico da pandemia.

### Limitações

Os resultados obtidos neste estudo refletem as práticas assistenciais de um centro de referência em estimulação cardíaca artificial, podendo ter sido influenciados pela expertise da equipe cirúrgica e pela infraestrutura especializada em assistência cardiovascular. Além disso, durante o período estudado, adaptações frequentes do atendimento e maior conhecimento do manejo da COVID-19 ocorreram com o passar das semanas epidemiológicas. Ainda não é possível medir o impacto do adiamento ou do cancelamento das operações eletivas ou, até mesmo, do fato de pacientes se absterem de ir ao hospital por medo de contrair a doença.

## Conclusões

Os dados do presente estudo mostram que as modificações assistenciais adotadas para o enfrentamento da pandemia da COVID-19 tiveram impacto no número e no perfil das cirurgias realizadas. Essas mudanças permitiram a realização segura dos procedimentos cirúrgicos durante a pandemia, mas não foram suficientes para evitar a transmissão intrahospitalar da COVID-19 e o seu impacto negativo na evolução de pacientes reconhecidamente de alto risco pela doença cardiovascular e comorbidades subjacentes.
