# Supplementary material for: Impacto da Pandemia por COVID-19 nos Procedimentos Cirúrgicos de Dispositivos Cardíacos Eletrônicos Implantáveis em um Centro de Referência Terciário
Source: Arq Bras Cardiol. 2021 Oct 6;117(4):765–9. [Article in Portuguese] doi: 10.36660/abc.20201378 (PMC8528374; doi:10.36660/abc.20201378)
Supplement: Translation

# Translation: Impact of the COVID-19 Pandemic on Cardiac Implantable Electronic Devices Procedures in a Tertiary Referral Center

**Authors:** Roberto Costa, Katia Regina da Silva, Sarah Caroline Martins Saucedo, Laisa Arruda Silva, Elizabeth Sartori Crevelari, Wagner Tadeu Jurevicius Nascimento, Thiago Gonçalves Silveira, Alfredo Fiorelli, Martino Martinelli, Fabio Biscegli Jatene

**Affiliations:** 1 Brasil Universidade de São Paulo Instituto do Coração - Unidade de Estimulação Elétrica e Marcapasso, São Paulo, SP – Brasil; 2 Brasil Universidade de São Paulo Faculdade de Medicina Hospital das Clinicas Instituto do Coração, São Paulo, SP – Brasil

**Keywords:** Defibrillators Implantable, Pacemaker, Artificial, Cardiac Pacing, Artificial, Pandemic, COVID-19, Hospitalization, Contamination, Quality of Health Care

## Introduction

The COVID-19 pandemic has caused a change in the surgical practice of many medical specialties worldwide.^1 - 4^ Medical societies have supported changes in cardiac pacing services routines and established new recommendations on the definition of case severity and urgency of surgical procedures.^5 , 6^

The aim of the present study was to evaluate the impact of the measures implemented in surgical procedures performed for artificial cardiac pacing during the pandemic. We compared the number of patients operated, clinical profile of patients, characteristics of procedures, and the rate of confirmed COVID-19 cases in the peak of the pandemic, and compared them with the period immediately before the pandemic.

## Methods

This was a prospective study, approved by the ethics committee Hospital das Clínicas da Faculdade de Medicina da Universidade de São Paulo (Plataforma Brasil: 26587419.7.0000.0068).

A total of 557 patients undergoing first implantation or reimplantation of cardiac implantable electrical devices (CIEDs) were evaluated. Specific data related to COVID-19 were included for analysis.

On March 21, 2020, the Instituto do Coração do Hospital das Clínicas da Faculdade de Medicina da Universidade de São Paulo (InCor-HCFMUSP) board established specific measures to be adopted for the fight against the COVID-19 pandemic – isolation of patients with COVID-19, postponement of elective surgeries, and prioritization of urgent life-saving procedures and those performed to prevent hemodynamic disturbances. For this purpose, surgeries were classified into urgent or elective ones ( Table 1 ), according to international recommendations.^5 , 6^

**Table 1:** – Classification of surgical procedures for implantation of cardiac electronic devices during the COVID-19 pandemic

Urgent procedures	Elective procedures
**First implantation**	**First implantation**
PM for irreversible advanced atrioventricular block Sinus node dysfunction with severe symptoms or long pauses	PM for non-advanced stable atrioventricular block Mild symptomatic sinus node disease
ICD for secondary prevention	ICD for primary prevention
CRT for severe refractory heart failure	CRT in stable patients
**Pulse generator replacement**	**Pulse generator replacement**
PM or ICD with minimum remaining battery life	PM or ICD with more than 6 weeks of remaining battery life
**Lead replacement**	**CIED upgrade**
Lead revision for malfunction in a PM dependent patient or ICD patient receiving inappropriate therapy	Upgrade for ICD only in case of secondary prevention Upgrade for CRT only in case of severe refractory heart failure
**Lead extraction**	**Lead extraction**
Treatment of CIED-related infection	Non-infected leads except when there is a need for obtaining venous access for lead replacement

*ICD: implantable cardioverter defibrillator; CIEDs: cardiac implantable electrical devices; PM: pacemaker; CRT: cardiac resynchronization therapy.*

The study patients were hospitalized after admission to the emergency department, by referral from other health care settings, or after analysis of the surgery waiting list. All surgeries were performed following routine surgical procedures. All patients were followed-up for 30 days after hospital discharge.

According to the time of surgery, patients were grouped into: Group 1 – before the pandemic (01 January – 20 March); Group 2 – peak of the pandemic (21 March – 31 July). Data were collected and organized using the REDCap software.^7^

The univariate analysis was used for comparison of surgical data during and before the pandemic, and significance level was set at 5%. Comparisons of means between the two periods were made using the Student’s t-test or the Mann Whitney test when assumption of data normality was not met. The chi-square test or the Fisher’s exact test was used to test the homogeneity of proportions.

## Results

Among the 557 patients, there was a similar distribution of men and women. Most patients were white (86.5%), and mean age was 64.5 ± 20.4 years. During the pandemic, there was a significant increase in the prevalence of patients with structural heart disease (p=0.016), arterial hypertension (p=0.047), atrial fibrillation (p=0.047), heart valve disease (p=0.048) and neoplasms (p=0.011) ( Table 2 ).

**Table 2:** – Clinical and surgical data of the study patients

Clinical and surgical data	Total (N= 557)	Group 1 Before the pandemic (N= 253)	Group 2 Pandemic peak (N= 304)	p
Male sex, n (%)	281 (50.4)	135 (53.4)	146 (48.0)	0.210
Age (years)	64.5 ± 20.4	65.6 ± 19.7	63.6 ± 20.9	0.248
Functional class (NYHA), n (%)				
I – II	453 (81.6)	212 (83.8)	241 (79.8)	0.378
III - IV	102 (18.4)	41 (16.2)	61 (20.2)	
Structural heart disease, n (%)				
Conduction or rhythm disturbance	204 (36.6)	109 (43.1)	95 (31.3)	0.016
Ischemic heart disease	101 (18.2)	48 (19.0)	53 (17.4)	
Non-ischemic heart disease	252 (45.2)	96 (37.9)	156 (51.3)	
Comorbidities, n (%)				
Arterial hypertension	360 (64.9)	153 (60.5)	207 (68.5)	0.047
Diabetes mellitus	151(27.2)	66 (26.1)	85 (28.2)	0.587
Atrial fibrillation	143 (25.8)	55 (21.7)	88 (29.1)	0.047
Heart valve disease	96 (17.3)	35 (13.8)	61 (20.2)	0.048
Chronic renal failure	59 (10.6)	25 (9.9)	34 (11.3)	0.600
Current or recent treatment for cancer	15 (2.7)	2 (0.8)	13 (4.3)	0.011
Procedures performed, n (%)				
First implantations	269 (48.3)	138 (54.6)	131 (43.1)	0.007
Reoperations	288 (51.7)	115 (45.4)	173 (56.9)	
Indication – First implants, n (%)				
Symptomatic bradyarrhythmia	202 (75.1)	107 (77.5)	95 (72.5)	0.533
Prophylaxis of sudden cardiac death	30 (11.2)	15 (10.9)	15 (11.5)	
Cardiac resynchronization	37 (13.8)	16 (11.6)	21 (16.0)	
Indication - Reoperations, n (%)				
Natural depletion of pulse generator	192 (66.7)	72 (62.6)	120 (69.4)	0.204
Lead malfunction	44 (15.3)	16 (13.9)	28 (16.2)	
CIED upgrade	25 (8.7)	11 (9.6)	14 (8.1)	
CIED infection	10 (3.5)	5 (4.3)	5 (2.9)	
Others	17 (5.9)	11 (9.6)	6 (3.5)	
Type of CIED, n (%)				
Ventricular pacemaker	76 (13.6)	21 (8.3)	55 (18.1)	0.007
Atrioventricular pacemaker	322 (57.8)	155 (61.3)	167 (54.9)	
Ventricular ICD	23 (4.1)	8 (3.2)	15 (4.9)	
Atrioventricular ICD	49 (8.8)	28 (11.1)	21 (6.9)	
Cardiac resynchronization therapy	82 (14.7)	37 (14.6)	45 (14.8)	
CIED removal	5 (0.9)	4 (1.6)	1 (0.3)	

*CIED: cardiac implantable electronic device; ICD: implantable cardioverter defibrillator.*

On average, 2.3 patients were operated per day during the peak of the pandemic, versus 3.2 patients/day in the period prior to the pandemic, representing a 27% reduction in the number of patients treated ( Figure 1 ). Also, during the pandemic, the type of procedures shifted to a predominance of reoperations (p=0.070) and increase in the rate of use of single-chamber pacemakers (p=0.007) ( Table 1 ).

**Figure 1:** – *Number of patients undergoing surgical procedures before and during the peak of the COVID-19 pandemic.*

In addition, during the pandemic peak, the length of hospital stay was shorter (p=0.001), with no difference in the rates of postoperative complications, readmissions, and mortality between the study groups ( Table 3 ).

**Table 3:** – Post-operative outcomes in patients undergoing implantation of cardiac implantable electrical devices before the COVID-19 and during the peak of the pandemic

Outcomes	Total (N= 557)	Group 1 Before the pandemic (N= 253)	Group 2 Pandemic peak (N= 304)	p
Time interval between hospital admission and surgical procedure (days)	5.4 ± 12.3	7.7 ± 15.3	3.5 ±8.7	<0.001
Time interval between surgical procedure and hospital discharge (days)	2.9 ± 7.3	3.5 ± 9.1	2.4 ± 5.4	<0.001
Length of hospital stay (days)	8.3± 16.6	11.2 ± 20.7	5.9 ± 11.8	<0.001
In-hospital death, n (%)	11 (2.0)	4 (1.6)	7 (2.3)	0.542
Thirty-day mortality, n (%)	7 (1.3)	2 (0.8)	5 (1.6)	0.367
Thirty-day rehospitalization, n (%)	16 (2.9)	9 (3.6)	7 (2.3)	0.377
Reoperation related to the CIED, n (%)	21 (3.8)	10 (4.0)	11 (3.6)	0.837
Confirmed diagnosis of COVID-19, n (%)	15 (2.7)	2 (0.8)	13 (4.3)	0.011

*CIED: cardiac implantable electrical device.*

Fifteen patients (2.7%) had a confirmed diagnosis of COVID-19. Two of them (0.4%) had been operated before the pandemic and were still hospitalized at the time of the study. Thirteen patients were operated during the pandemic; 6 (1.1%) had a confirmed diagnosis of COVID-19 during hospitalization, and 7 (1.3%) during the 30-day follow-up.

All-cause mortality occurred in 18 (3.2%) patients, with no significant difference between the groups ( Table 3 ). Of the 15 patients with COVID-19, seven died, and COVID-19 was the primary cause in all of them. The other deaths were caused mainly by cardiovascular diseases (n=7; 1.3%), followed by infection unrelated to CIEDs (n=2; 0.4%), surgical complications (n=1; 0.2%) and advanced cancer (n=1; 0.2%).

## Discussion

The present study showed that changes in health care routines allowed a safe and effective management of patients with indications for cardiac pacing surgical procedures during the COVID-19 pandemic. However, these changes caused a reduction in the number of patients undergoing CIED implantation for the first time and in the complexity of the CIEDs used, and a relative increase in replacements of pulse generator due to battery depletion.

Despite the greater severity of patients seen during the pandemic, there was a significant reduction in the length of hospital stay, due to a reduction in both preoperative and postoperative periods. On the other hand, no significant differences were found in the types of CIED-related complications, need for surgical reinterventions or rehospitalization rate within 30 days after discharge between the two periods analyzed or as compared with the historical profile of the institution.^8^

Unfortunately, the measures adopted could not prevent patients from being infected with the new coronavirus, which was observed in 2.7% of the study patients. Supply shortages impacting COVID-19 testing, as reported previously,^9^ as well as the non-detection of patients already infected with SARS-CoV-2 at admission may have contributed to the under-notification of cases. At least two patients who had been operated before the pandemic, with prolonged hospital stay, had COVID-19. Among those operated during the pandemic, six patients had manifestations of COVID-19 during hospitalization. In the other seven, diagnosis of COVID-19 was made during the 30-day period after discharge, and thus we cannot know whether the patient was infected during the pandemic or after hospital discharge.

Although not statistically significant, overall mortality of patients operated during the pandemic was higher as compared with those operated before the pandemic and influenced by the death of seven patients (out of 15) who developed COVID-19 during the pandemic. The high rate of comorbidities in the COVID-19 patients was determinant for the unfavorable outcome of this group.

Now, post-pandemic recommendations for a safe return to general activities are being discussed.^10^ The experience here reported may serve as a basis for actions that should be taken during a possible new pandemic peak.

### Limitations

This study was conducted in a referral center for artificial cardiac pacing procedures, and the possibility that our results may have been influenced by the expertise of the surgery team and specialized facilities cannot be ruled out. Also, during the study period, adaptations in health care services and greater knowledge of the management of COVID-19 occurred over the epidemiological weeks. So far, it has not been possible to measure the impact of postponement or cancellation of elective surgeries, or of the fact that patients have avoided going to the hospital because of the fear of getting infected with the new coronavirus.

## Conclusions

The present study showed that the health care measures adopted to face the COVID-19 pandemic had an impact on the number and the types of surgeries performed. Although these changes allowed safe surgical procedures during the pandemic, they did not prevent patients from contracting COVID-19 and the negative impact of this disease on the course of patients known to be at high risk due to cardiovascular diseases and underlying comorbidities.
